# Respiratory resistance and reactance in adults with sickle cell anemia: Correlation with functional exercise capacity and diagnostic use

**DOI:** 10.1371/journal.pone.0187833

**Published:** 2017-12-08

**Authors:** Cirlene de Lima Marinho, Maria Christina Paixão Maioli, Jorge Luis Machado do Amaral, Agnaldo José Lopes, Pedro Lopes de Melo

**Affiliations:** 1 Biomedical Instrumentation Laboratory—Institute of Biology and Faculty of Engineering, and BioVasc Research Laboratory—Institute of Biology, State University of Rio de Janeiro, Rio de Janeiro—Brazil; 2 Department of Hematology—Faculty of Medical Sciences, State University of Rio de Janeiro, Rio de Janeiro—Brazil; 3 Department of Electronics and Telecommunications Engineering, State University of Rio de Janeiro, Rio de Janeiro, Brazil; 4 School of Medical Sciences, Pulmonary Function Testing Laboratory, Rio de Janeiro/RJ, State University of Rio de Janeiro, Rio de Janeiro–Brazil; 5 Rehabilitation Sciences Post-Graduation Program, Augusto Motta University Centre, Rio de Janeiro, Brazil; Université Claude Bernard Lyon 1, FRANCE

## Abstract

**Background:**

The improvement in sickle cell anemia (SCA) care resulted in the emergence of a large population of adults living with this disease. The mechanisms of lung injury in this new population are largely unknown. The forced oscillation technique (FOT) represents the current state-of-the-art in the assessment of lung function. The present work uses the FOT to improve our knowledge about the respiratory abnormalities in SCA, evaluates the associations of FOT with the functional exercise capacity and investigates the early detection of respiratory abnormalities.

**Methodology/Principal findings:**

Spirometric classification of restrictive abnormalities resulted in three categories: controls (n = 23), patients with a normal exam (n = 21) and presenting pulmonary restriction (n = 24). FOT analysis showed that, besides restrictive changes (reduced compliance; p<0.001), there is also an increase in respiratory resistance (p<0.001) and ventilation heterogeneity (p<0.01). FOT parameters are associated with functional exercise capacity (R = -0.38), pulmonary diffusion (R = 0.66), respiratory muscle performance (R = 0.41), pulmonary volumes (R = 0.56) and airway obstruction (R = 0.54). The diagnostic accuracy was evaluated by investigating the area under the receiver operating characteristic curve (AUC). A combination of FOT and machine learning (ML) classifiers showed adequate diagnostic accuracy in the detection of early respiratory abnormalities (AUC = 0.82).

**Conclusions:**

In this study, the use of FOT showed that adults with SCA develop a mixed pattern of respiratory disease. Changes in FOT parameters are associated with functional exercise capacity decline, abnormal pulmonary mechanics and diffusion. FOT associated with ML methods accurately diagnosed early respiratory abnormalities. This suggested the potential utility of the FOT and ML clinical decision support systems in the identification of respiratory abnormalities in patients with SCA.

## Introduction

Sickle cell disease (SCD) encompasses a group of conditions that cause red cells to become sickle-shaped. Sickle cell anemia (SCA) is the most common and often the most severe kind of SCD, corresponding to a monogenic, recessive genetic condition that results in changes in the structure of the red blood cells and repercussions in various organs. Worldwide, this disease affects approximately 300,000 children annually and is considered one of the most prevalent disorders among the group of existing hereditary diseases [1–3].

As a result of a dramatic improvement in SCD care over the last decades, life expectancy has improved significantly, with an observed median survival of more than 60 years [3]. The emergence of a larger population of adults living with SCA necessitates further understanding of the overall changes in their respiratory function. Understanding the mechanisms of lung injury may guide choices in the development of new therapies and clinical care.

SCA causes involvement in several organs, especially the lungs, which are frequently affected in this disease through acute thoracic syndrome (ATS). In addition to being a major cause of death and the second largest cause of hospital admission in SCA, ATS correlates with pulmonary wheezing and cognitive dysfunction in these patients, resulting from ischemia and stroke caused by vaso-occlusion of the capillaries that irrigate the brain tissue [4–9]. Therefore, early diagnosis of ATS is fundamental for reversing unfavorable clinical outcomes [4].

Traditional tests of pulmonary function allow us to detect the presence of obstructive, restrictive or mixed changes [10]. However, to perform these exams, it is necessary for the patient to understand and perform a forced expiratory maneuver to obtain reliable results [11]. In the particular case of SCA, the performance of these tests may be difficult due to the presence of cognitive deficiency, resulting in the underdiagnosis of pulmonary changes in a timely manner and compromising adequate follow-up and treatment of these patients [12].

Initially described by Dubois et al. [13], the forced oscillation technique (FOT) is a simple exam that requires little cooperation on the part of patients. This may be particularly important in patients with SCA, in whom the cognitive deficiency may be so high that this may be the only feasible exam. A large research effort has been developed in our laboratory to improve the clinical use and technology used in FOT-based exams. Among the main results obtained are the early identification of the effects of smoking [14], sarcoidosis [15], rheumatoid arthritis [16], silicosis [17], systemic sclerosis [18], cystic fibrosis [19] and asbestos-exposed workers [20]. These results provide evidence that FOT can contribute to the simplification of respiratory assessments in patients with SCA to elucidate the pathophysiological mechanism of ATS, as well as the early detection of these respiratory abnormalities. Although this method presents a high potential to improve respiratory evaluations in SCA, only one study in the literature has focused on the use of FOT in patients with SCA [21]. The cited work, however, was limited to analyze the association between obstructive defects and ATS.

Despite several attractive characteristics of the FOT, this method has not been widely introduced into clinical practice [22]. It happens, at least in part, because the physiological or clinical meaning of the derived parameters is not clear. The six-minute walk test (6MWT) reflects the ability to perform the activities of daily living and exhibits a satisfactory correlation with the maximal O_2_ consumption. Therefore, the 6MWT has the potential to elucidate the physiological and clinical meaning of FOT parameters.

Another factor that may prevent the wide use of this method in clinical practice is that, in the context of a diagnostic framework, the interpretation of resistance and reactance curves and the derived parameters requires training and experience, and it is a difficult task for the untrained pulmonologist. Machine learning (ML) methods are increasingly used in medicine [23–25], and may contribute to simplify the interpretation of FOT results and to enhance the diagnostic accuracy. Promising results were obtained in previous studies of our group in the detection of the early effects of smoking [26], the diagnosis [27] and classification of the severity [28] of chronic obstructive pulmonary disease, and airway obstruction in asthma [29]. To the best of our knowledge, however, there is no study evaluating the diagnostic potential of the FOT, and developing clinical decision support systems to facilitate the diagnostic use and to improve the accuracy of this method in patients with SCA.

Based on the abovementioned considerations, the objectives of the present study were (1) to deepen our knowledge about the changes in ventilatory mechanics in patients with SCA by evaluating the resistive and reactive properties obtained through FOT; (2) to examine the association between these findings and changes in diffusing capacity, respiratory muscles performance and functional exercise capacity; and (3) to investigate the clinical potential of FOT in the early diagnosis of respiratory abnormalities in patients with SCA, including the development of a clinical decision support system.

## Methods

This is an observational study comprised of an evaluation of prevalent cases, in which the evaluation unit was the individual. The experimental phase of this research was developed at the Department of Pulmonology of the Pedro Ernesto University Hospital (HUPE) and at the Biomedical Instrumentation Laboratory of the State University of Rio de Janeiro. This study was approved by the Research Ethics Committee of the Pedro Ernesto University Hospital, and Informed Consent Form was obtained from all volunteers and patients. The protocol of this study obeys the Declaration of Helsinki and was registered at ClinicalTrials.gov (identifier: NCT02565849).

### Subjects

The control group consisted of individuals over 18 years of age, with no history of smoking and respiratory diseases, an absence of cardiac dysfunction, orthopedic impairment, and spirometric examination and of the forced oscillation technique compatible with normality.

**Fig 1:**
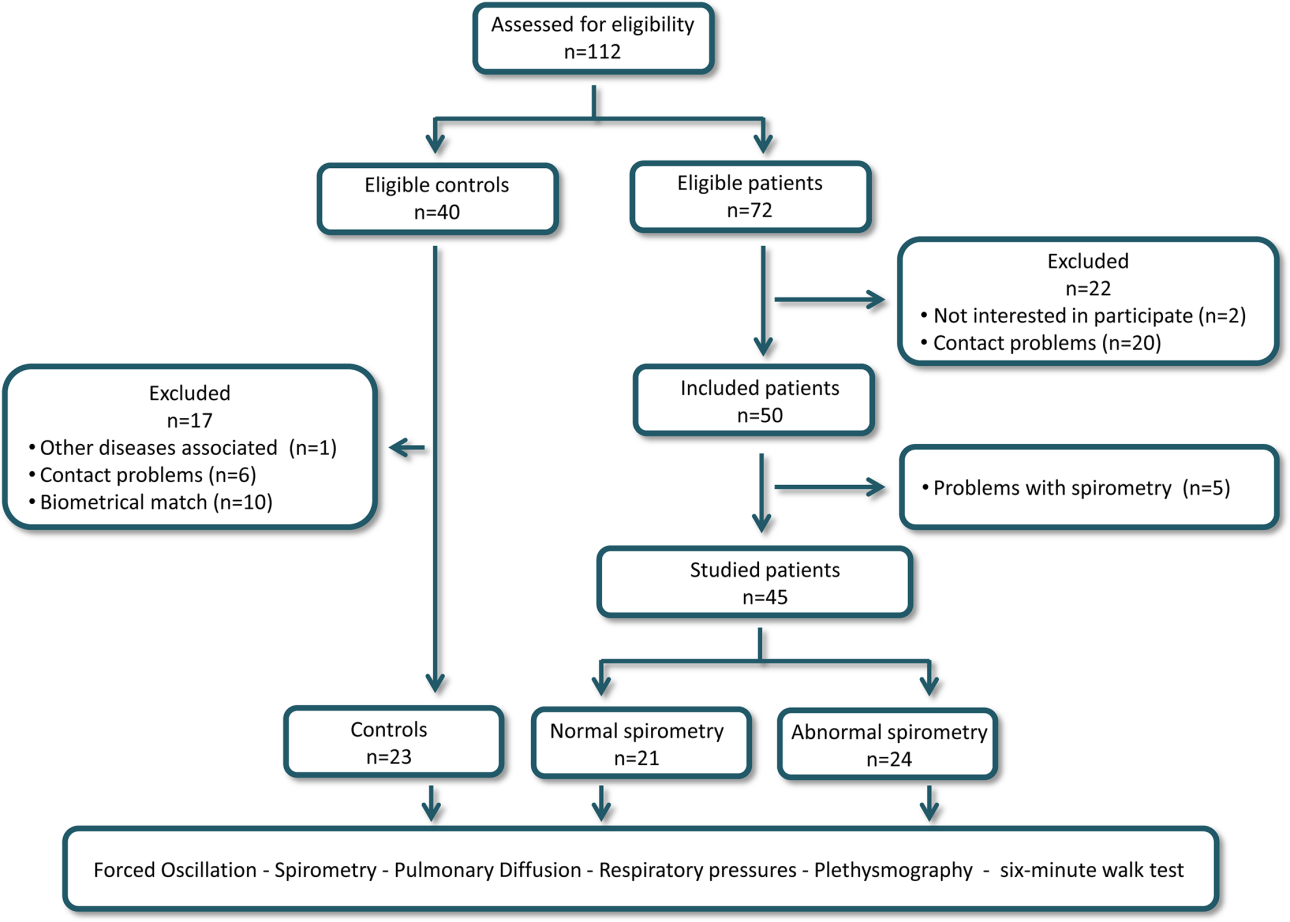
Study flowchart describing the examined volunteers and the sequence of the performed analysis.

Patients older than 18 years with homozygous expression of hemoglobin S (type SS) were included in the group of patients and were followed up at the outpatient clinic of the Hematology Department of Pedro Ernesto University Hospital. The exclusion criteria were other hemoglobinopathies, absence of history of respiratory infections and hospitalization in the previous three months, asthma, smoking, diabetes, rheumatologic and oncological diseases, de-compensated heart disease, acute pain and inability to perform the forced oscillations and pulmonary function tests. The flowchart of the study is described in Fig 1.

### Forced oscillation technique

During the FOT examinations, the volunteers remained seated, with their heads in the neutral position and used a nasal clip. They were instructed to maintain basal breathing throughout the examination period through a silicone mouthpiece, with the cheeks held up by their hands to reduce the shunt effect. The system used applies sinusoidal pressure signals in the frequency range of 4 to 32 Hz. The instrument has previously been described in detail [30], and the technique followed international standards [31]. A pseudorandom sinusoidal signal with a 2 cmH_2_O peak-to-peak amplitude was applied by a loudspeaker. The pressure input was measured with a Honeywell 176 PC pressure transducer (Microswitch, Boston, MA, USA), and the airflow with a screen pneumotachometer coupled to a similar transducer with a matched frequency response. The signals were digitized at a rate of 1024 Hz for periods of 16 s by a personal computer, and a fast Fourier transform was computed using blocks of 4096 points with 50% overlap. A minimal coherence function of 0.9 was considered adequate. Three measurements were obtained, and the final results of the test were calculated as the mean of these three measurements.

The interpretation of the respiratory impedance data and its association with the structural properties of the respiratory system was performed using FOT parameters. The resistive parameters were obtained from the linear extrapolation of the real impedance component between 4 and 16 Hz, allowing analysis of the slope of the resistance dependence with frequency (S). This parameter reflects frequency-dependent changes in airflow distribution, describing the homogeneity of the resistance distribution in the lung. It is related to both spatial and temporal inhomogeneity in the distribution of the gas flow within the system [32]. In healthy subjects, this parameter presents values ​​close to zero, becoming progressively more negative in individuals with pulmonary involvement [19, 33]. This analysis also allowed us to attain the intercept resistance (R0) and average resistance (Rm). Resistances measured between 4 and 16 Hz are related to the airway and tissue Newtonian resistance in addition to the delayed airway resistance resulting from the gas redistribution. In this context, R0 estimates how the cited properties work at low frequencies [32]. Rm, on the other hand, is associated with the airway caliber [34]. The resistance of the respiratory system at 4 Hz (R4) was also included in the performed analysis.

The reactive data were interpreted using the mean reactance (Xm) [35] and resonance frequency (fr) [32]. The inertial component of reactance presents a positive phase (pressure precedes airflow), and the compliant component shows a negative phase (pressure that is delayed in relation to the airflow). The resonant frequency occurs when both components contribute equally to respiratory reactance, and the phase between pressure and flow are equal to zero. These parameters reflect changes in airway heterogeneity as well as tissue changes. The respiratory system dynamic compliance (Cdyn) [36] was also studied. Cdyn reduced values are associated with decreased lung compliance and/or increased airway resistance [10]. The total mechanical load to be overcome by the respiratory muscles to promote the movement of air in the respiratory system, including the resistive and elastic effects, was evaluated using the absolute value of the respiratory impedance (Z4) [34, 37].

### Spirometry

Analyses of spirometry, plethysmography, and pulmonary diffusion capacity were performed at the Pulmonary Function Test Laboratory at Pedro Ernesto University Hospital and obeyed the standard protocols of the American Thoracic Society/European Respiratory Society [38, 39] and the recommendations of the Brazilian Consensus of Spirometry [40]. The analyzed parameters were the forced expiratory volume in the first second (FEV_1_), forced vital capacity (FVC), the FEV_1_/FVC ratio, and the forced expiratory flow (FEF) between 25% and 75% of the FVC (FEF/FVC) ratio. These parameters were expressed as absolute values and as a percentage of the predicted values (% of predicted), and the reference values were obtained from the equations of Pereira et al. [41] Forced expiratory maneuvers were repeated until three sequential measurements were obtained. The studied indexes were obtained using the better curve, which was selected based on the higher values of FEV_1_ plus FVC. The software automatically detected non-acceptable maneuvers according to ATS criteria, providing the quality control of the spirometric exams.

### Plethysmography

Plethysmography exams were performed with a constant volume and variable pressure plethysmograph (HD CPL nSpire Health Ltd., Hertford, UK). The evaluated parameters were the total lung capacity (TLC), functional residual capacity (FRC) and residual volume (RV), as well as their relationships (RV/TLC and FRC/TLC). Airway resistance (Raw) was also measured. The reference values were based on the equations described by Neder et al. [42].

### Pulmonary diffusion capacity

During these measurements, the individual remains in the seated posture and receives instructions for the proper performance of the respiratory maneuver. Patients with sickle cell anemia were submitted to routine hematology exams through venipuncture. Diffusion was corrected for the concentration level of hemoglobin values obtained from these examinations, which were performed on the same day of the pulmonary function test. This analysis included the following parameters: carbon monoxide diffusion capacity (DLCO), alveolar volume (AV) and diffusion coefficient (DLCO/VA).

### Respiratory muscle pressure analysis

Using the same instrument applied in plethysmographic measurements, respiratory muscle strength was evaluated by the maximal inspiratory pressure (PImax) obtained starting from the residual volume, and the maximum expiratory pressure (PEmax) measured from total lung capacity. PImax and PEmax were the highest values obtained after three measures [43].

### Functional exercise capacity

The 6MWT was performed on a flat surface in a 30-meter corridor. These exams followed the recommendations of the American Thoracic Society [44]. Heart rate, blood pressure, grade of dyspnea (modified Borg scale), respiratory rate and oxygen saturation (SpO_2_) were measured before and immediately after the end of the test. The predicted values were calculated using the equations for the Brazilian population described by Britto et al. [45].

### Clinical decision support system

The choice of the appropriate classifier algorithms was performed evaluating seven different methods: Support Vector Machine with Linear Kernel, Adaboost with Decision Tree Classifiers, K Nearest Neighbor, Random Forests, Support Vector Machine with Radial Basis Kernel (SVMR) and the Parzen Classifier. Many of these classifiers have been applied with a great deal of practical success in respiratory research [46–49]. A detailed description of them can be found in previous studies [26–29, 50]. Because the data set size was relatively small, the performance evaluation was analyzed using k-fold cross-validation, which makes better use of the available dataset [26–29].

### Data processing, presentation and statistical analysis

Statistical analyses were performed using Origin® 8.0 (Microcal Software Inc., Northampton, Massachusetts, United States). The results are present as the mean±SD. Initially, the sample distribution characteristics were assessed using Shapiro-Wilk’s test. A one-way ANOVA with Tukey’s test was performed to analyze the normally distributed data; conversely, a non-parametric analysis (Kruskal-Wallis) with a Mann-Whitney test was performed for the non-normally distributed data. Differences with p≤0.05 were considered statistically significant.

In normal distributions, the correlations were studied using Pearson`s correlation coefficient, while Spearman’s correlation was used in non-normal distributions. These correlations were classified as follows [51]:

- Small or no correlation: between 0 and 0.25 (or -0.25);
- Reasonable correlation: between 0.25 and 0.50 (or -0.25 to -0.50);
- Moderate to good correlation: between 0.50 and 0.75 (or -0.50 to -0.75);
- Very good to excellent correlation: greater than 0.75 (or -0.75).

Considering that several correlations were computed, we performed a correction in the significance level to minimize the chances of making a Type I error. We used a modified Bonferroni approach, which requires dividing usual p-value by an estimate of the effective number of independent correlations used [52]. As FOT describes resistive and reactive properties, two independent variables were considered. In general, four independent variables are observed in other exams, which results in eight independent correlations and a corrected significance level for correlation analysis of 0.0063 (0.05/8).

A receiver operation characteristic (ROC) analysis was used to evaluate the clinical potential of the FOT indexes in the detection of respiratory alterations. The values of sensitivity, specificity, and area under the curve (AUC) were obtained based on the optimal cut-off point, as determined by the ROC curve analysis. According to the literature, ROC curves with AUCs between 0.50 and 0.70 indicate low diagnostic accuracy, AUCs between 0.70 and 0.90 indicate moderate accuracy, and AUCs between 0.90 and 1.00 indicate high accuracy [53, 54]. Goedhart et al. [55] considered 0.7 to be a good cut-off value for a useful discriminator for clinical use. In the present study, we considered 0.75 to be the minimum value of the AUC for adequate diagnostic accuracy. The ROC analyses were conducted using MedCalc 12 (MedCalc Software, Mariakerke, Belgium). This part of the study follows the STARD requirements for studies of diagnostic accuracy [56].

The sample size was calculated based on the criteria of the comparison of the area under a ROC curve with a null hypothesis value. The aim was to show that an AUC of 0.75, describing adequate diagnostic accuracy [55], was significantly different from the null hypothesis value of 0.5, which indicates no discriminating power. This analysis was performed using MedCalc 13 (MedCalc Software, Mariakerke, Belgium), according to the theory described by Hanley and McNeil [57]. A type I error of 0.10 and a type II error of 0.10 were assumed as adequate, which resulted in a minimum of 20 volunteers per group.

## Results

Table 1 shows the clinical, biometrical and spirometric characteristics of the studied individuals. With the exception of FEV_1_/FVC, all of the spirometric values showed significantly reduction in patients with SCA (ANOVA p<0.05).

**Table 1:** Clinical, biometric and spirometric characteristics of control individuals and patients with sickle cell anemia.

	Control (n = 23)	Sickle Cell Anemia NE (n = 21)	Sickle Cell Anemia AE (n = 24)	NE-AE p	ANOVA p
Biometric					
Age (years)	32.6 ± 8.9	34.9 ± 17.8	27.9 ± 9.3	-	ns
Body mass (kg)	62.4 ± 7.0	62.8 ± 10.6	59.3 ± 7.5	-	ns
Height (cm)	164.0 ± 0.1	165.0 ± 0.1	165.0 ± 0.1	-	ns
BMI (kg/m^2^)	23.2 ± 1.6	23.0 ± 3.6	21.8 ± 2.4	-	ns
Gender (M/F)	3 / 20	12 / 9	11 / 13	-	-
Clinical data					
VOC up to 5 years	-	15	6	-	-
VOC after 5 years	-	6	18	-	-
HT	-	9	9	-	-
AH		5	1	-	
HALY > 1	-	18	22	-	-
HALY < 1	-	3	2	-	-
Stroke	-	2	5	-	-
Articular pain	-	7	9	-	-
Spirometry					
FVC (L)	3.7±0.6	3.5±0.7	2.9±1.0**	ns	0.01
FVC (%)	101.2±9.7	92.8±13.6	72.8±14.1***	0.0001	0.0001
FEV_1_ (L)	3.9±0.5	2.9±0.6	2.3±0.7***	0.01	0.0001
FEV_1_ (%p)	98.4±10.0	91.1±16.1	68.0±13.7***	0.0001	0.0001
FEV_1_/FVC	82.6±6.0	82.4±5.0	79.6±7.5	ns	ns
FEV_1_/FVC(%)	97.1±6.2	97.6±5.1	92.1±6.8*	0.01	0.05
FEF max (L)	7.1±2.0	5.7±2.2	5.2±1.8**	ns	0.05
FEF max (%)	105.1±18.3	78.5±28.8***	67.0±15.9***	ns	0.0001

Data are means±SD. Sickle Cell Anemia NE = Patients with normal spirometric exams; Sickle Cell Anemia AE = Patients with altered spirometric exams; VOC: vaso-occlusive crisis; HT: hydroxyurea therapy; AH: arterial hypertension; HALY: Hospital admissions in the last year; % = Percentage of the predicted values; ns = not significant (p >0.05)

*p<0.05 related to control group

**p<0.01 related to control group

*** p<0.0001 related to control group.

The results obtained in the analysis of plethysmography and pulmonary diffusion are described in Table 2. These analyses showed significant reductions in TLC (p<0.05), FRC (p<0.05) and in the RV/TLC (%) (p<0.005). Raw increased significantly among the studied groups (p<0.0001).

**Table 2:** Plethysmography, pulmonary diffusion and respiratory muscle pressure values in the studied groups.

	Control (n = 23)	Sickle Cell Anemia NE (n = 21)	Sickle Cell Anemia AE (n = 24)	NE-AE p	ANOVA p
Plethysmography					
TLC (L)	5.1±0.9	4.9±0.8	4.3±1.2*	ns	0.05
TLC (%)	96.0±8.89	89.1±8.0**	74.6±10.5****	0.0001	0.0001
FRC (L)	2.3±0.6	2.2±0.6	1.9±0.7*	ns	<0.05
FRC (%)	78.9±16.4	73.0±12.4	60.1±13.9****	<0.05	0.0001
RV (L)	1.3±0.4	1.4±0.4	1.2±0.4	ns	ns
RV (%)	81.2±23.2	92.6±20.5	73.9±30.8	ns	ns
RV/TLC	25.5±6.3	29.2±7.8	29.9±8.8	ns	ns
RV/TLC (%)	85.5±20.9	100.8±20.9	108.4±27.5***	ns	0.005
Raw (cmH2O/L/s)	1.9±0.9	4.3±1.8****	4.4±1.7****	ns	0.0001
Pulmonary diffusion					
DLCOabs	26.1±4.5	20.2±5.9****	19.0±6.1****	0.0001	0.0001
DLCOabs (%)	110.9±11.1	77.9±15.6****	71.3±16.9****	0.0001	0.0001
DLCOcorr	26.1±4.5	24.8±7.4	22.8±8.4	ns	ns
DLCOcorr (%)	110.9±11.1	95.2±18.9*	84.8±23.9****	0.0001	0.0001
DLCO/AVcorr	5.32±0.6	5.4±1.1	6.0±1.3	ns	ns
DLCO/AVcorr (%)	120.2±13.1	97.7±5.1	126.6±30.9	ns	ns
AV (L)	4.9±0.9	4.6±1.0	3.8±1.2***	0.005	0.01
AV (%)	92.6±7.6	82.5±9.0****	67.9±12.1****	0.0001	0.0001
Respiratory pressures					
MIP	-82.4±30.3	-47.8±65.1	-62.4±47.9	ns	ns
MIP (%)	86.7±23.9	74.0±33.4	68.2±24.7	ns	ns
MEP	114.1±35.9	81.7±30.1***	80.9±29.9***	ns	0.005
MEP (%)	68.2±15.2	46.7±19.1****	44.7±11.6****	ns	0.0001

Data are means±SD. Sickle Cell Anemia NE = Patients with normal spirometric exams; Sickle Cell Anemia AE = Patients with altered spirometric exams; % = Percentage of the predicted values; ns = not significant (p >0.05)

*p<0.05 related to control group

**p<0.01 related to control group

***p<0.001 related to control group

****p<0.0001 related to control group.

Pulmonary diffusion analyses revealed significant reductions considering the uncorrected diffusion values (DLCOabs, DLCOabs (%)) (p<0.0001). Significantly reduced indexes were even observed in individuals without detected spirometric alterations (p<0.0001). Similar reductions were observed in AV (%). When a correction for a hemoglobin examination was applied, DLCOcorr(%) was significantly reduced (p<0.0001).

The analysis of the respiratory pressures showed significant reductions of the expiratory values p<0.005), while inspiratory pressures did not show significant differences.

**Fig 2:**
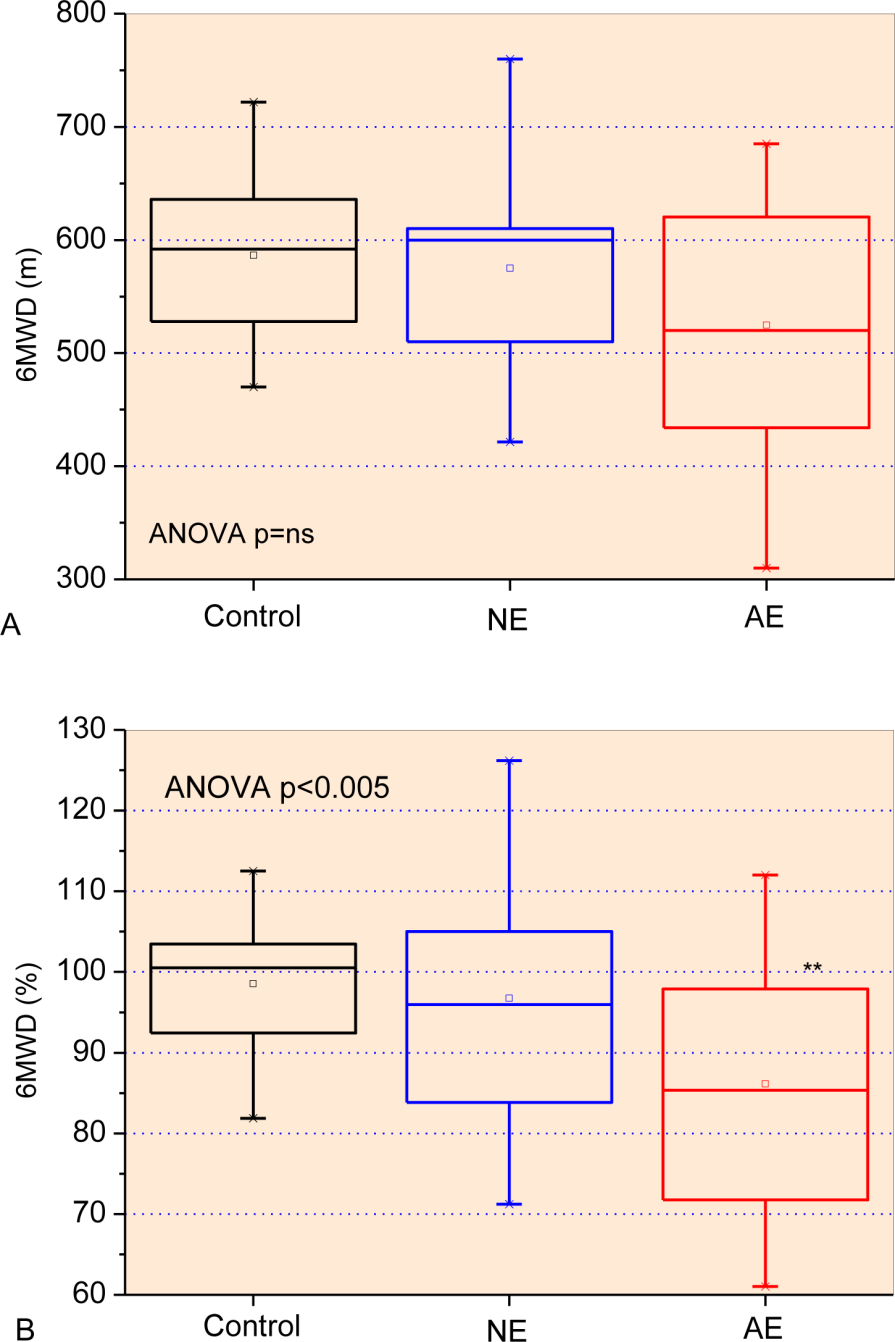
Functional exercise capacity analysis describing the six-minute walk distance (A) predicted percentage (B), initial (C) and final (D) Borg Scale among the studied groups. Associated parameters are presented in Table 3. The top and the bottom of the box plot represent the 25th- to 75th-percentile values, while the circle represents the mean value, and the bar across the box represents the 50th-percentile value. The whiskers outside the box represent the 10th-to 90th-percentile values. **p<0.01 related to control group.

The functional exercise capacities among the studied groups are described in Fig 2, while the associated parameters are described in Table 3. The increase in airway obstruction resulted in a significant reduction in the Post-6MWT heart rate (p<0.005), Pre- and Post-6MWT diastolic blood pressure (p<0.01), and Pre- and Post-6MWT SpO2 (%) (p<0.0001). Increased Pre- and Post-6MWT respiratory rate (p<0.005), Pre- and Post-6MWT systolic blood pressure (p<0.05), and Final Borg Scale (p<0.001) were also observed.

**Table 3:** Parameters associated with the functional exercise capacity among the studied groups.

	Control (n = 23)	Sickle Cell Anemia NE (n = 21)	Sickle Cell Anemia AE (n = 24)	NE-AE p	ANOVA p
Pre-6MWT heart rate (beats/min)	81.2±10.7	78.9±11.7	83.6±12.5	ns	ns
Post-6MWT heart rate (beats/min)	102.1±16.6	93.6±15.9	95.8±16.4**	ns	0.005
Pre-6MWT respiratory rate (breaths/min)	16.8±3.1	19.2±2.9*	20.8±4.8***	ns	0.005
Post-6MWT respiratory rate (breaths/min)	20.5±3.3	22.5±3.0	25.0±6.2**	ns	0.005
Pre-6MWT systolic blood pressure (mm Hg)	113.9±13.5	124.8±15.5*	115.7±13.0	ns	0.05
Post-6MWT systolic blood pressure (mm Hg)	121.2±11.7	140.2±15.6****	126.2±13.5	0.01	0.0001
Pre-6MWT diastolic blood pressure (mm Hg)	77.1±11.5	82.0±13.9**	70.3±12.0	0.01	0.01
Post-6MWT diastolic blood pressure (mm Hg)	84.9±10.3	87.0±10.5	74.5±14.2*	0.005	0.001
SpO_2_ (%) Pre-6MWT	97.7±1. 5	96.0±2.5**	93.8±3.1****	0.05	0.0001
SpO_2_ (%) Post-6MWT	97.8±1.2	95.5±3.2**	93.2±3.8****	0.05	0.0001

Data are means±SD. Sickle Cell Anemia NE = Patients with normal spirometric exams; Sickle Cell Anemia AE = Patients with altered spirometric exams; 6MWD: Six-minute walk distance; (%): predicted percentage. CF: Cardiac frequency. RR: Respiratory rate. SBP: Systolic blood pressure. DBP: Diastolic blood pressure. SpO_2_: Peripheral oxygen saturation. ns = not significant (p >0.05)

*p<0.05 related to control group

**p<0.01 related to control group

***p<0.001 related to control group

****p<0.0001 related to control group.

Fig 2 shows the mean curves describing the resistance and reactance values for each studied group. Airway obstruction in SCD introduced a significant increase in resistance (Fig 2A; ANOVA p<0.0001). Comparing these curves, patients with normal spirometry showed a slight and non-significant increase of resistance (Fig 2A; p = ns. Significantly higher values of resistance were observed in patients with abnormal spirometry (3.91±0.57 cmH_2_O/L/s) in comparison with controls (2.77±0.23 cmH_2_O/L/s; p<0.0001). Although visually clear, Sickle Cell Disease do not introduced statistically significant changes comparing the mean Xrs curves (Fig 2B).

**Fig 3:**
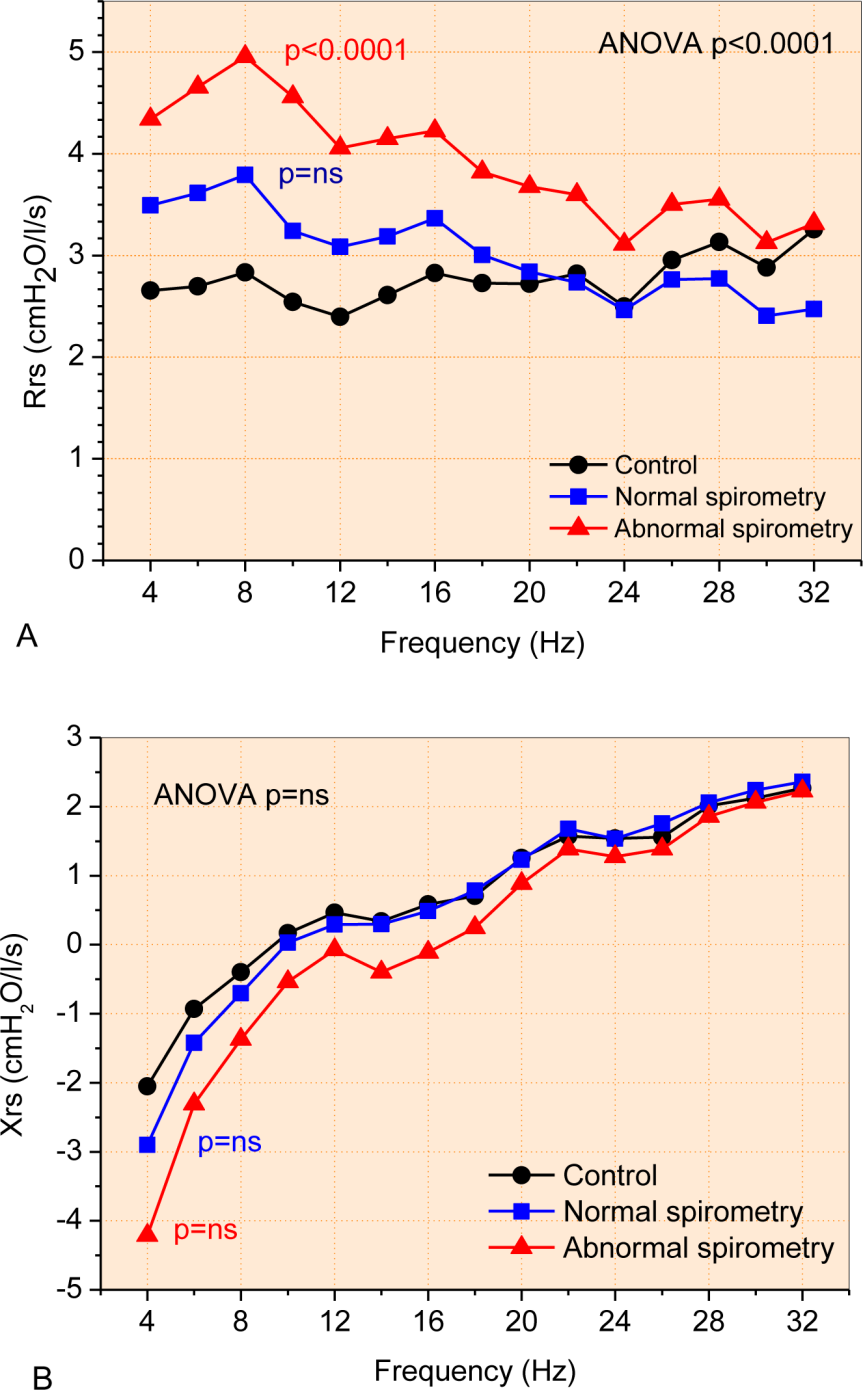
Changes in mean respiratory resistance (A) and reactance (B) curves as a function of frequency in patients with SCA.

The increase of respiratory involvement in SCD resulted in higher values of total resistance (Fig 3A, p<0.001), airway resistance (Fig 2B, p<0.01) and resistance at 4 Hz (Fig 2C, p<0.01). The homogeneity of ventilation was reduced (Fig 2D, p<0.01).

Considering the reactive properties, significant reductions in Xm (Fig 3A, p<0.05) and Cdyn (Fig 3B, p<0.0001) were observed, as well as a significant increase in resonance frequency (Fig 3B, p<0.0005) and impedance modulus (Fig 3D, p<0.005).

FOT parameters were not correlated with FEV_1_/FVC and FEFmáx(%), while S, Xm and Fr were not correlated with spirometric indexes. The strongest correlations were presented by Cdyn, showing good and direct (positive) correlation with FEV_1_ (L) and FVC (L) (Table 4). In general, the degree of association among FOT and spirometric parameters were reasonable to good.

**Table 4:** Correlation analysis among forced oscillation parameters and spirometric results. The strongest correlation for each FOT parameter is described in bold.

	FEV_1_ (L)	FEV_1_ (%)	FVC (L)	FVC (%)	FEF máx (L)
R0	**-0.62**	-0.39	-0.59	-0.44	-0.45
	<0.0001	<0.001	<0.0001	<0.0001	0.0001
Rm	**-0.63**	-0.41	-0.61	-0.44	-0.43
	<0.0001	0.0005	<0.0001	<0.0001	<0.0005
R4	**-0.58**	-0.31	**-0.58**	-0.36	-0.34
	<0.0001	ns	<0.0001	<0.005	<0.005
Cdyn	**0.79**	0.50	0.77	0.54	0.36
	<0.0001	<0.0001	<0.001	<0.0001	<0.005
Z4	**-0.67**	-0.46	-0.64	-0.49	-0.30
	<0.0001	<0.0001	<0.0001	<0.0001	ns

Xm was not correlated with plethysmographic parameters and RV was not correlated with FOT indexes. Similar to the analysis performed with spirometry, the correlations among FOT and plethysmographic parameters were reasonable to good (Table 5). It was interesting to note that, also similar to the analysis performed using spirometric parameters, the strongest correlations among the FOT parameters were presented by Cdyn, showing good and direct correlation with TLC (L).

**Table 5:** Correlation analysis among forced oscillation parameters and volumetric results. The strongest correlation for each FOT parameter is described in bold.

	TLC (L)	TLC (%)	FRC (L)	FRC (%)	RV/TLC	RV/TLC (%)	Raw
R0	-0.45	-0.39	-0.48	-0.42	0.39	0.26	**0.54**
	<0.0001	<0.001	<0.0001	<0.0005	<0.001	ns	<0.0001
Rm	-0.43	-0.41	**-0.49**	-0.43	0.40	0.26	0.47
	<0.0005	<0.001	<0.0001	<0.0005	<0.001	ns	<0.0001
R4	-0.47	-0.33	-0.47	-0.39	0.37	0.23	**0.49**
	<0.0001	ns	<0.0001	<0.001	<0.001	ns	<0.0001
S	0.29	0.12	0.18	0.14	-0.13	-0.13	**-0.51**
	ns	ns	ns	ns	ns	ns	<0.0001
Fr	-0.18	**-0.36**	-0.27	-0.32	-0.01	0.09	0.04
	ns	<0.005	ns	ns	ns	ns	ns
Cdyn	**0.56**	0.45	0.54	0.48	-0.55	-0.39	-0.42
	<0.0001	0.0001	<0.001	<0.0001	<0.0001	<0.001	<0.0005
Z4	-0.35	-0.41	-0.45	-0.42	**0.56**	0.36	0.39
	<0.005	0.0005	<0.0001	<0.0005	<0.0001	<0.005	<0.001

Considering the associations between FOT and pulmonary diffusion, it may be observed that the correlations were among reasonable to good values (Table 6). FOT parameters were not correlated with DLCO/AV and S was not correlated with diffusion parameters. Resistive parameters and Z4 presented inverse (negative) good associations with DLCOa, while Xm and fr presented reasonable degree of association with AV (%). Similar to that observed in previous correlation analyses with spirometry (Table 4) and plethysmography (Table 5), the strongest correlations among FOT parameters were presented by Cdyn, which showed a good association with AV (L).

**Table 6:** Correlation analysis among forced oscillation parameters and pulmonary diffusion capacity results. The strongest correlation for each FOT parameter is described in bold.

	DLCOa	DLCOa (%)	DLCOc	DLCOc (%)	DLCO/AVc	DLCO/AVc (%)	AV (L)	AV (%)
R0	**-0.62**	-0.49	-0.45	-0.31	0.06	0.06	-0.55	-0.51
	<0.0001	<0.001	0.0001	ns	ns	ns	<0.0001	<0.0001
Rm	**-0.62**	-0.49	-0.48	-0.34	0.01	0.02	-0.56	-0.52
	<0.0001	0.0005	<0.0001	<0.005	ns	ns	<0.0001	<0.0001
R4	**-0.57**	-0.38	-0.42	-0.24	0.10	0.13	-0.53	-0.43
	<0.0001	0.001	<0.0005	ns	ns	ns	<0.0001	<0.0005
Xm	0.11	0.23	0.10	0.27	-0.09	0.02	0.18	**0.35**
	ns	ns	ns	ns	ns	ns	ns	<0.005
Fr	-0.13	-0.24	-0.11	-0.28	0.12	-0.002	-0.19	**-0.35**
	ns	ns	ns	ns	ns	ns	ns	<0.005
Cdyn	0.63	0.45	0.53	0.40	-0.09	-0.06	**0.66**	0.53
	<0.0001	0.0001	<0.001	<0.001	ns	ns	<0.0001	<0.0001
Z4	**-0.57**	-0.43	-0.48	-0.36	0.01	0.06	**-0.57**	-0.53
	<0.0001	<0.0005	<0.0001	<0.005	ns	ns	<0.0001	<0.0001

The relationships among FOT and respiratory pressure parameters are presented in Table 7. Resistive parameters (R0, Rm and R4) were reasonably associated with Pe, while S, Xm, fr and Z4 were not associated with respiratory pressures. Similar to previous correlation analysis, Cdyn presented the highest correlation, showing a good association with Pe (R = 0.41).

**Table 7:** Correlation analysis among forced oscillation parameters and respiratory muscle pressure. The strongest correlation for each FOT parameter is described in bold.

	Pi	Pi (%)	Pe	Pe (%)
R0	0.33	-0.25	**-0.38**	-0.23
	<0.005	ns	<0.001	ns
Rm	0.29	-0.21	**-0.36**	-0.20
	ns	ns	<0.005	ns
R4	0.37	-0.26	**-0.39**	-0.21
	<0.005	ns	<0.001	ns
Cdyn	-0.24	0.24	**0.41**	0.28
	ns	ns	0.0005	ns

Table 8 describes the associations between the FOT and functional exercise capacity. Fr and R4 do not presented significant correlations, while R0 presented a direct reasonable degree of association with the Final Borg Scale and final RR. Rm was correlated with Final Borg Scale. Reasonable associations were also observed with the initial RR (S), initial and final SpO_2_ (Xm), while Cdyn and Z4 presented significant associations with six minute walking test distance 6MWTD. Z4 showed the highest degree of association among FOT and the functional exercise capacity parameters (R = -0.38).

**Table 8:** Correlation analysis among forced oscillation parameters and 6MWT results. The strongest correlation for each FOT parameter is described in bold.

	6MWT	6MWT(%)	RRInitial	RR Final	SpO_2_Initial	SpO_2_ Final	BorgScaleInitial	BorgScaleFinal
R0	-0.23	-0.05	0.30	**0.35**	-0.11	-0.17	0.16	**0.35**
	ns	ns	ns	<0.005	ns	ns	ns	<0.005
Rm	-0.28	-0.10	0.24	0.30	-0.11	-0.16	0.16	**0.37**
	ns	ns	ns	ns	ns	ns	ns	<0.005
S	-0.09	-0.18	**-0.35**	-0.33	-0.04	0.09	-0.08	-0.07
	ns	ns	<0.005	ns	ns	ns	ns	ns
Xm	0.17	0.18	-0.15	-0.04	0.35	**0.37**	-0.08	-0.20
	ns	ns	ns	ns	<0.005	<0.005	ns	ns
Cdyn	**0.35**	0.13	-0.28	-0.30	0.16	0.21	-0.17	-0.20
	<0.005	ns	ns	ns	ns	ns	ns	ns
Z4	**-0.38**	-0.19	0.20	0.22	-0.15	-0.25	0.09	0.25
	<0.001	ns	ns	ns	ns	ns	ns	ns

The ROC analyses for the FOT parameters are described in Table 9. It was interesting to observe that S presented adequate diagnostic accuracy even in the absence of changes in spirometry (AUC>0.75). Similar analyses in patients with abnormal spirometric exams showed that R0, R4, S and Z4 presented AUC values adequate for diagnostic use and that Cdyn was more accurate among the FOT parameters (AUC = 0.79). LOOCV analyses in these parameters resulted in reduced values of AUC, such that none of the FOT parameters achieved an appropriate value for clinical use (AUC>0.75).

**Table 9:** Evaluation of the diagnostic accuracy of the forced oscillation parameters in detecting respiratory alterations in patients with sickle cell anemia and normal and abnormal spirometric exams. Values of area under the curve (AUC), sensitivity (Se), specificity (Sp) for the optimal cut-off points obtained using receiver operating characteristic (ROC) curves.

	R0	Rm	R4	S	Xm	fr	Cdyn	Z4
Normal exam								
AUC	0.67	0.63	0.62	**0.77**	0.62	0.69	0.66	0.63
Se (%)	61.90	52.38	57.14	66.67	57.14	66.67	61.9	57.14
Sp (%)	60.87	56.52	52.17	60.87	60.87	65.22	60.87	56.52
Cut-off	3.07	3.02	2.93	-14.67	0.66	10.53	0.02	3.83
Abnormal exam								
AUC	**0.78**	0.74	**0.75**	**0.76**	0.73	0.73	**0.79**	**0.78**
Se (%)	79.17	66.67	66.67	70.83	66.67	62.5	75	75
Sp (%)	73.91	69.57	65.22	69.57	65.22	60.87	73.91	73.91
Cut-off	3.23	3.33	3.26	-15.69	0.5	10.51	0.01	4.22

The analysis performed in the classifier algorithms revealed that the Parzen Classifier associated with an exhaustive search of the best forced oscillation parameters was the more accurate in patients with normal spirometry (AUC = 0.82; Se = 85.7% and Sp = 78.3%; Fig 6). The SVMR classifier (without an exhaustive search) was the more discriminative in conditions of abnormal spirometry (AUC = 0.86, Se = 90.5%, Sp = 73.9%; Fig 6). Both classifiers achieved an appropriate value for clinical use (AUC>0.75).

## Discussion

The primary findings of the present study were as follows: 1) adult patients with SCA present increased respiratory resistance, reduced dynamic compliance, increased heterogeneity in the distribution of the resistive and reactive properties and increased total impedance. 2) FOT parameters are correlated with functional exercise capacity decline and pulmonary diffusion changes in adults with SCA; and 3) a combination of FOT and ML methods provided adequate detection of early respiratory abnormalities in these patients.

There were no significant differences in body mass, height or age among the studied groups (Table 1). In general, the spirometric parameters were highest in normal subjects and decreased significantly in groups of patients with SCA. Of the 42 individuals with sickle cell anemia examined, 57% had abnormalities in lung function, of which 87% were restrictive. According to Miller et al. [58], this behavior is associated with the occurrence of repeated episodes of ATS, which develop areas of local scarring. In line with this proposition, it was observed that patients with worse pulmonary function had more recent vaso-occlusive crisis than the group with normal spirometry (Table 1).

In agreement with the presence of a restrictive disease, the pulmonary volumes were reduced and the RV/TLC ratio showed a significant increase (Table 2). In addition, Raw was significantly elevated (p<0.0001) in patients with SCA, both with normal and altered spirometry (Table 2). These alterations can be attributed to the pulmonary sequelae acquired with ATS episodes, the adoption of a superficial respiratory pattern due to chronic chest pain and to functional changes in ventilatory biomechanics in face of pain episodes [59, 60].

Klings et al. carried out diffusion measurements in patients with SCA [59], reporting findings with and without correction for hemoglobin. The authors describe that, even after the correction, the diffusion remained reduced, and this result was associated with disruption of the alveolar-capillary membrane, impairing gas exchange. In the present study, similar results were observed considering the absence of correction for hemoglobin (DLCOa and DLCOa(%) in Table 2). However, when performing the correction (DLCOc), these results showed a tendency to reduce but without statistical significance (p = 0.07), which disagrees with the cited study. In a recent study in children with sickle cell disease [61], the measurement of pulmonary diffusion to corrected carbon monoxide for hemoglobin also showed no significant difference. This behavior was associated with the compensatory mechanism of dilation of the pulmonary capillaries, secondary to increased blood volume, aiding diffusion. Table 2 shows that, when evaluating the predicted value to the DLCOc, the reduction was statistically significant (p<0.0001). AV was significantly reduced, which may be explained by the reduction in the available exchange area due to the usual episodes of ATS and ventilation-perfusion imbalance in this population [58, 62].

The expiratory muscle pressure of patients with SCA showed a significant reduction, a condition not shown by the inspiratory muscles (Table 2). Previous studies have found similar results in patients with SCA and other lung disease [63–65]. The hypothesis considered is that the expiratory muscles demonstrate greater susceptibility to vaso-occlusive episodes compared to inspiratory muscles and this is due to the local anatomical circulatory conditions. The diaphragm presents an extensive network of collateral capillaries that supply the need for oxygen, due to its uninterrupted muscular work. This vascular pattern provides the diaphragm less likely to suffer damage resulting from hypoxemia [63, 66, 67]. Repeated episodes of vaso-occlusive crisis may result in muscle necrosis in patients with SCA. It has previously been indicated that during repeated painful crisis, it is possible that the destruction of expiratory muscle fibers is more frequent, favoring their lower contraction potential [68–70].

The 6MWT distance showed a significant reduction in the functional exercise capacity of patients with SCA and abnormal spirometry (Fig 2). Similar results were found in previous studies [71, 72]. The metabolic and cardiopulmonary derangement caused by the disease itself justifies the low test performance, associated with the lower VO_2_ peak obtained through the cardiopulmonary exercise test [71]. The parameters associated with the functional exercise capacity (Table 3) presented changes in close agreement with the involved pathophysiology, describing increased respiratory rate, systolic blood pressure, and decreased values of Post-6MWT heart rate, diastolic blood pressure and SpO_2_. These results may reflect a reduced capacity for circulatory adaptation during the activity, in addition to a higher propensity to develop pulmonary artery hypertension, which are common in these patients [73].

The results described in Fig 2A and Fig 3 indicate that, in addition to the known restrictive characteristics, SCA also introduces changes in homogeneity and respiratory obstruction. Arteta et al. have recently described that children with SCA have frequent wheezing and bronchial hyperreactivity of multifactorial causes [62]. The predominantly obstructive respiratory pattern was identified at this stage. A possible explanation for this occurrence is the increased pulmonary capillary blood flow, secondary to chronic anemia, which generates greater cardiac output and dilation of the pulmonary veins, thus narrowing the smaller caliber airways. As a result, there is limited expiratory airflow and respiratory obstruction [11, 62]. It is now thought that this pattern is perpetuated in adults [62]. Reports of patients with SCA presenting asthma in childhood are quite common and some authors link the pulmonary obstructive pattern to this finding [74]. The finding that patients with SCA present asthma already has an important impact on morbidity and mortality in this group of patients. Cook and colleagues studied children and observed a predominantly restrictive respiratory pattern using spirometry [9]. Other authors highlight that recurrent wheezing in some patients is predominantly related to chronic pulmonary inflammation due to the presence of inflammatory biomarkers resulting from intravascular hemolysis. This finding could justify the perpetuation of respiratory obstruction and wheezing even when treatment with bronchodilators is ongoing [5, 75].

**Fig 4:**
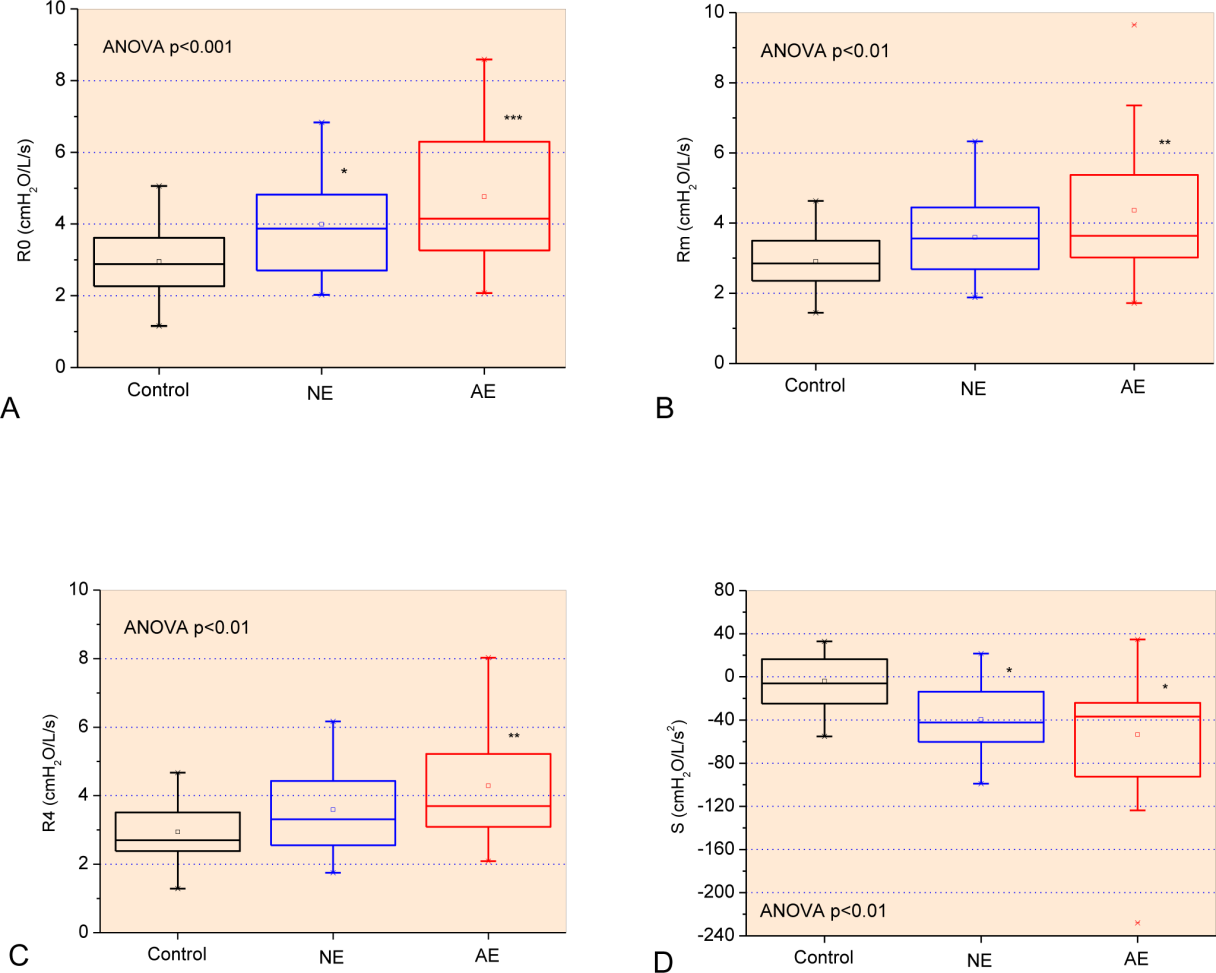
Influence of changes in airway obstruction in resistive parameters obtained from the control group and patients with normal (NE) and abnormal (AE) spirometry: Respiratory system resistance (R0; Fig A), mean resistance (Rm, Fig B), resistance in 4 Hz (R4; Fig C) and slope of respiratory resistance (S; Fig D). *p<0.05 related to control group; **p<0.01 related to control group; ***p<0.001 related to control group.

In SCA, R0 increased significantly, even in patients with normal spirometry (Fig 4A). This demonstrates that, even at the stage where pulmonary dysfunction was not detected by spirometry, there is an increase in the total resistance of the respiratory system. Patients with altered spirometry had even higher values ​​of R0. SCA introduces increased pulmonary capillary diameter by increased circulating blood flow. For this reason, extravasation of circulating fibrocytes occurs in the parenchyma, which favors tissue thickening [11]. These results agree with previous studies, which state that SCA introduces resistive pulmonary changes secondary to repeated episodes of ATS [62, 76–81]. Increased resistance is also justified by the greater presence of collagen fibers in the pulmonary parenchyma resulting from remodeling of air spaces, airways and interstitial fibrosis. We found only one work in the literature that used FOT to evaluate adult patients with SCA [21]. The authors indicated that in patients who suffered major episodes of ATS, R0 was shown to be higher, characterizing an obstructive profile, in agreement with our findings.

Rm and R4 also demonstrated elevated values in SCA groups (Fig 2B and 2C). In childhood, it is common for asthma to be present in these individuals and thus one of the first changes detected is pulmonary obstruction. In adulthood, the most common dysfunction in these individuals is pulmonary restriction. From the data provided by FOT, we observed that in individuals with abnormal spirometric tests, the resistances were increased. This finding indicates that individuals with sickle cell anemia also present obstruction at more advanced ages, suggesting a mixed pattern [62, 82–84]. In line with this proposition, Table 4 describes the presence of good and inverse correlations of R0, Rm and R4 with spirometric parameters associated with obstruction and restriction, FEV_1_ and FVC (L), respectively. In addition, the correlation between R0, Rm and R4 and plethysmographic parameters (Table 5) showed a good inverse correlation with FRC and direct correlation with Raw. Wedderburn and colleagues found results in this direction in children with SCD [61]. The cited study showed increased resistance even after bronchodilator testing. The authors suggest that increased resistance may be associated with the compensatory mechanism of increased blood flow. This introduce dilation of the pulmonary veins, and increased cardiac output secondary to chronic anemia, as these changes in the structure of the capillaries compress the distal alveoli, contributing to the pulmonary obstruction (44). In close agreement with this hypothesis, Table 6 shows inverse associations among R0, Rm and R4 and pulmonary diffusion. An increase in the O_2_ flow from the atmosphere to the mitochondria is crucial for a normal response to exercise. This may explain the results observed in Table 8, in which increased R0, Rm and R4 values are associated with reduced functional exercise capacity of SCA patients.

Fig 4D shows a significant reduction of S in both patients with normal spirometry and with altered spirometry. These results indicate that the initial respiratory changes in SCA result in alterations in the homogeneity of the resistive properties of the respiratory system and that these changes increases with respiratory restriction. In agreement with this interpretation, S presented a reasonable and direct correlation with FEFmax (L) (Table 4), and a good inverse association with Raw (Table 5). The reasonable direct association with absolute DLCO (Table 6) is also consistent with this hypothesis.

**Fig 5:**
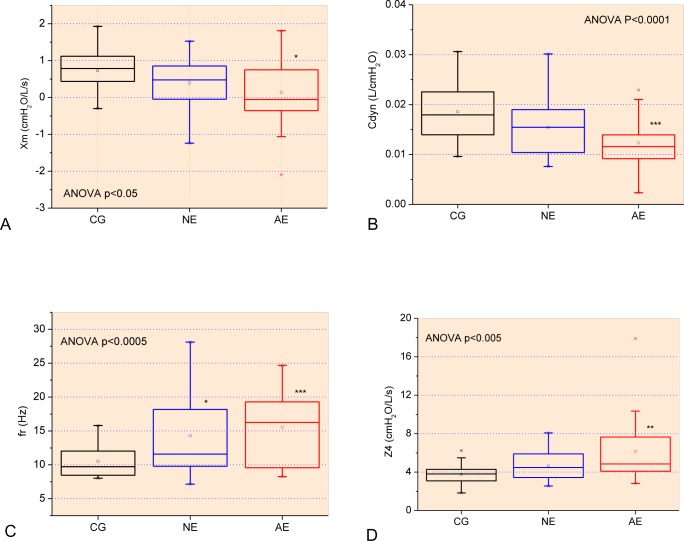
Comparative analysis of the reactive parameters obtained from the control group and patients with normal (NE) and abnormal (AE) spirometry: mean respiratory reactance (Xm; Fig A), resonant frequency (fr, Fig B), dynamic compliance (Cdyn; Fig C) and respiratory impedance module in 4Hz (Z4; Fig D). *p<0.05 related to control group; **p<0.01 related to control group; ***p<0.001 related to control group. *p<0.05 related to control group; **p<0.01 related to control group; ***p<0.001 related to control group.

In line with the results observed in S, the average Xm value became more negative with increased respiratory limitations in SCA patients (Fig 5A), while fr was significantly elevated (Fig 5C). These parameters presented reasonable correlations with FEV_1_ and FVC (%) (Table 4), as well as with plethysmographic parameters associated with restriction (TLC (%) and FRC, Table 5). These changes are associated with a reduction in the homogeneity of the reactive properties of the respiratory system [18]. This may be associated with the presence of tissue non-homogeneities, which are in line with the correlations observed with diffusion parameters (Table 6). Another important factor is the low compliance due to repeated episodes of ATS, which results in more negative values of mean reactance. These changes introduce important changes in the gas distribution. Similar results were found in patients with Cystic Fibrosis and Systemic Sclerosis, where Xm was more negative in individuals with the disease compared to healthy subjects [18, 19].

Cdyn showed a significant reduction with the presence of the restrictive pattern (Fig 5B). This is in agreement with the increase in fr, described in Eq 1, and the involved pathophysiology.

$$fr=\frac{1}{2\pi \sqrt{IC}}$$ (1)

where I and C are associated with dynamic inertance [19] and compliance [10], respectively. This reduction is associated with the increase in respiratory system elastance and the reduction in dynamic compliance, due to a reduced number of functioning small airways. In line with this interpretation, Cdyn showed the strongest correlations among the FOT and spirometric parameters (Table 4), showing very good and direct correlation with FEV_1_ (L) and FVC (L). Cdyn also presented the strongest correlations among FOT parameters and plethysmography, showing good associations with parameters related with restrictive abnormalities (Table 5). It is worth noting that, similar to that observed in previous correlation analyzes with spirometry (Table 4) and plethysmography (Table 5), the strongest correlations among FOT parameters and diffusion capacity analysis was presented by Cdyn (Table 6). This provides evidence that this parameter is able to adequately describe the physiological changes in patients with SCA. These abnormal changes can be attributed to the repeated episodes of ATS suffered by these patients, as well as to parenchymal, airway and chest changes. Because of the predisposition to embolic events due to hemocyte dysmorphism, these patients are subject to events of bone infarction that may compromise the adequate biomechanical movement of the costal gradient and, consequently, lead to atelectasis [85]. These results may also be associated with thickening of the peripheral and central airways secondary to inflammation, extravasation of fibrocytes and collagen deposition for local repair [80]. Since FOT is a simple and noninvasive technique, we can suppose that the Cdyn may contribute to the analysis of the elastic properties of the respiratory system in individuals with SCA, offering an alternative to invasive measures using an esophageal balloon [86].

The total impedance modulus of the respiratory system increased with spirometric changes (Fig 5D), and presented reasonable to good correlations with spirometric and plethysmographic indexes of airway obstruction and restriction (Tables 4 and 5). This demonstrates that FOT was able to describe the increase in energy expenditure to overcome the elastic and resistive components involved in the ventilatory process of these patients. In addition to the justifications presented previously for resistive and reactive alterations, we can add the presence of vasoconstriction of the pulmonary arteries resulting in an imbalance in ventilation-perfusion ratio and increase in dead space. Thus, larger areas of pulmonary atelectasis [4] are introduced, which may contribute considerably to increase the impedance modulus. These new information describe an increase in respiratory work, which contributes to the understanding of the limitation to exercise present in these patients. In general, the results presented in Figs 3 to 5 suggest the presence of a pattern of mixed respiratory disorder in SCA.

The moderate to good correlations observed between FOT and respiratory diffusion (Table 6) may be explained by two mechanisms: (1) when airflow resistance is raised, the ventilation of the alveoli is impaired. As a consequence, the PO_2_ gradient in the alveoli is reduced, resulting in diminished diffusion velocity and reduced gaseous exchange, and (2) restrictive pulmonary disease causes a fall in pulmonary volumes. The loss of functioning lung tissue reduces the area of diffusion and in this way impairs gaseous exchange. Note that FOT provides parameters associated with airflow resistance (R0, Rm, R4, S) and lung compliance (Cdyn, fr, Z4). Thus, the association between FOT and diffusion capacity in SCA is expected, since FOT provides parameters closely related with gaseous exchange impairment mechanisms.

The decrease in exercise capacity in individuals with SCD arises as a result of ventilatory limitation, abnormalities in gas exchanges, and cardiovascular dysfunction. Abnormal changes affecting primarily the lungs will also have significant systemic effects. This may explain the reasonable and significant associations between FOT and exercise capacity observed in Table 8. This indicates that the ventilatory changes evaluated by FOT may contribute to anticipate the limitations during exercise, which happens due to the causal relationships between adequate ventilation, oxygen availability and physical performance.

**Fig 6:**
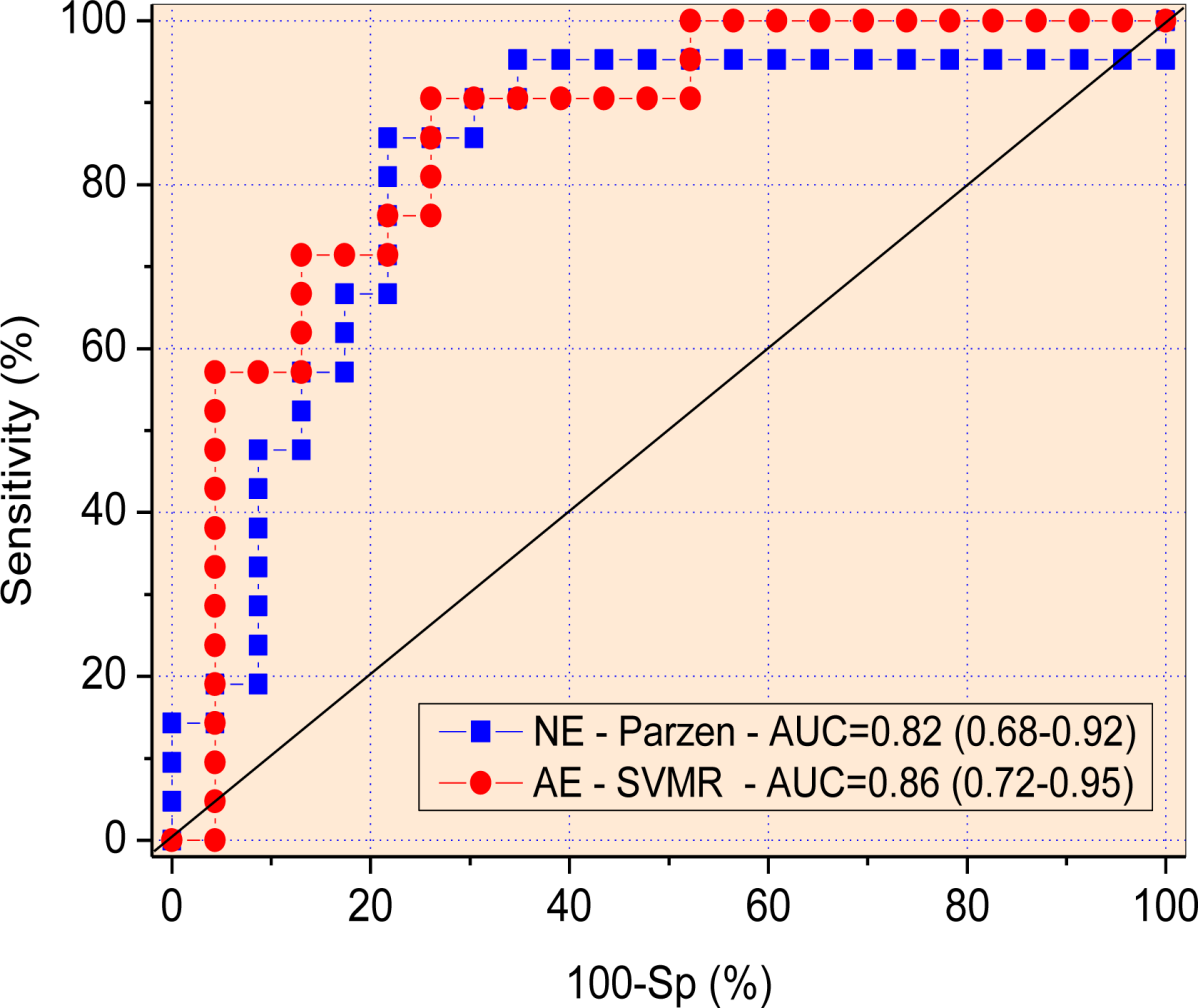
ROC curves, AUCs and the 95% confidence interval for the most accurate machine learning algorithm associated with FOT measurements in patients with normal exams (A), and with abnormal spirometric exams (B).

There is currently a consensus in the literature on the need to develop new sensitive and noninvasive lung-function testing to allow early and accurate detection of pulmonary function decline [87, 88]. The presence of significant changes in the group with normal spirometry (Figs 4–6) provides evidence that FOT could contribute in this issue. ROC analysis performed in these parameters were evaluated considering AUCs > 0.75 to be a good cut-off value for a useful discriminator for clinical use [53–55]. In this study, S was able to detect early abnormal changes in SCA, even in the presence of normal spirometric exams. In patients with abnormal spirometry, the accuracy of this parameter do not increased due to the presence of a higher variability (Fig 4). Five FOT parameters presented adequate AUC values in this group. However, none of these parameters reached adequate values of diagnostic accuracy when considering the more restrictive criteria obtained using LOOCV. In contrast, the use of a ML clinical decision support system allowed us to achieve adequate diagnostic accuracy in patients with normal spirometry (Fig 6). This interesting finding suggests that FOT associated with ML resulted in a highly sensitive analysis able to detect initial decline in lung function of patients with SCA. This analysis was also adequate to identify respiratory changes in patients with abnormal spirometric exams (Fig 6), which provides additional evidence of the usefulness of this analysis in diagnostic purposes. These results are in close agreement with recent studies in which the use of ML clinical decision support systems improved the diagnostic accuracy in chronic obstructive pulmonary disease [27] and asthma [29].

The present study had some limitations, including its relatively small sample size. This limitation was minimized by the LOOCV procedure, but it is still a limitation in this study, and future studies should include a larger number of subjects.

The generalizability of the results of the present study to other populations is unknown since the study was conducted in a Brazilian population at a single practice site. Future research should therefore be addressed in multicenter studies to expand the generalizability of these findings. It is important to consider, however, that the experimental conditions of the present study enhanced its generalizability. The study was performed in a typical setting under usual clinical procedures, and it was used broad inclusion criteria. Interested readers may examine the demographic characteristics and inclusion and exclusion criteria adopted to evaluate if they are likely to obtain similar outcomes in their own patient population.

This study primarily focused on patients with hemoglobin SS to exclude possible confounding factors regarding severity and clinical profile and because this is the most common and often most severe kind of SCD. However, many other types of SCD should be considered because they exhibit many disparate features, including different structural changes within the respiratory system. The development of studies in these disorders is a clear direction for future research.

## Conclusion

This study initially improved our knowledge regarding the respiratory abnormalities in patients with SCA providing a detailed analysis of the changes in resistance and reactance in these patients. In the second stage of the study, it was shown that FOT parameters (Cdyn, R0, Z4) are associated with functional exercise capacity, pulmonary diffusion, respiratory muscle performance, pulmonary volumes and airway obstruction. Finally, it was observed that a combination of FOT and ML methods accurately detected early respiratory abnormalities in patients with SCA. This suggested the potential utility of the FOT associated with ML clinical decision support systems in the analysis of the respiratory abnormalities in patients with SCA.
